# Elevated interleukin-25 and its association to Th2 cytokines in systemic lupus erythematosus with lupus nephritis

**DOI:** 10.1371/journal.pone.0224707

**Published:** 2019-11-07

**Authors:** Malarvili Selvaraja, Maha Abdullah, Masita Arip, Voon Kin Chin, Anim Shah, Syafinaz Amin Nordin

**Affiliations:** 1 Department of Pathology, Faculty of Medicine and Health Sciences, Universiti Putra Malaysia, Serdang, Selangor, Malaysia; 2 Allergy and Immunology Research Centre, Institute for Medical Research, Jalan Pahang, Kuala Lumpur, Wilayah Persekutuan Kuala Lumpur, Malaysia; 3 School of Biosciences, Taylor’s University, No. 1, Jalan Taylor’s, Subang Jaya, Selangor, Malaysia; 4 Department of Medicine,Faculty of Medicine and Health Sciences, Universiti Putra Malaysia, Serdang, Selangor, Malaysia; 5 Department of Medical Microbiology & Parasitology, Faculty of Medicine and Health Sciences, Universiti Putra Malaysia, Serdang, Selangor, Malaysia; University of Missippi Medical Center, UNITED STATES

## Abstract

Systemic lupus erythematosus (SLE) is an autoimmune disorder that is associated with lupus nephritis, initiated by the deposition of immune complexes in the kidney; subsequently, this induces the overexpression of cytokines. Lupus nephritis is known as one of the major clinical manifestations that affect the disease severity in SLE patients. An increased number of resident periglomerular and immune cells in the kidney has the potential to affect the equilibrium of different immune cell subsets, such as Th1, Th2, Th17, and Tregs, which may be central to the induction of tissue damage in kidney by exerting either proinflammatory or anti-inflammatory effects, or both. This equilibrium has yet to be confirmed, as new players such as IL-25 remain undiscovered. IL-25 is a cytokine of the IL-17 family, which stimulates Th2-mediated immune response when overly expressed. Thus, the aim of this research is to determine the plasma levels of IL-25 and Th2-associated cytokines (IL-4, IL-5, IL-6, IL-9, IL-10, IL-13) in SLE patients with (SLE-LN) and without lupus nephritis. Sixty-four (n = 64) SLE patients and fifteen (n = 15) healthy individuals were recruited. This study demonstrated that the IL-9, IL-10 and IL-25 had significantly increased expressions in SLE-LN, followed by SLE without LN, compared to healthy controls. Meanwhile, IL-5 and IL-6 had significantly reduced. No significant difference was observed with IL-13, while the level of IL-4 was undetectable. Furthermore, IL-9 and IL-10 were significantly correlated with the IL-25, and IL-25, IL-9 and IL-10 were positively correlated with the disease severity score, SLEDAI. In conclusion, IL-25 and its associated Th2 cytokines (IL-9 and IL-10) may be involved in SLE pathogenesis. These cytokines could be potential biomarkers in monitoring and predicting the disease severity during SLE pathogenesis.

## Introduction

Systemic Lupus Erythematosus (SLE) is one of the most complex polygenic autoimmune disorders with diverse immune-pathological abnormalities and clinical manifestations that vary between individuals. Among the clinical manifestations, lupus nephritis (LN) is the most common and serious organ-associated complications of SLE that impose severe impact on a patient’s survival. Previous study has reported that Asian SLE patients have higher prevalence of lupus nephritis than Caucasian SLE patients do [1]. In addition, Asian SLE patients with renal involvement were noted to have more severe features and lower probability of long-term survival compared to American and European patients [2].

Generally, more than one-third of SLE patients with lupus nephritis experience basic clinical signs and symptoms, such as weight gain, dark urine, swelling around the eyes, legs, ankles, or fingers, and high blood pressure. Typically, lupus nephritis will generate abnormal urinalysis result with increased serum creatinine level, persistent proteinuria of more than 0.5 grams per day, low level of glomerular filtration rate, C3 and C4, presence of blood cells and/or casts in urine, and high erythrocyte sedimentation rate (ESR) [3]. Moreover, the increased levels of blood urea nitrogen and anti-dsDNA studies also support the indication of LN. On top of that, uncontrolled lupus nephritis may lead to progressive loss of kidney function [4].

The key pathogenesis of SLE involves aberrant immune reactions against endogenous nuclear antigens, leading to the production of autoantibodies. A substantial reduction in clearance activity and formation of immune complexes will subsequently lead to local inflammation and tissue or organ damage [5]. These events may indirectly regulate the expression of soluble mediators, such as cytokines, chemokines, complement proteins, innate and adaptive responses [6], and portray an imbalance in T helper cell (Th) cytokines and other circulating chemokines. Additionally, the progression of SLE in patients with LN could be due to the overexpression of cytokines. In LN patients, activated T cells induce proinflammatory cytokines production, which activate neutrophils and macrophages [7]. On the other hand, Th1-, Th2- and Th17-associated cytokines have robust positive correlations with the SLEDAI [8]. Several studies have reported on the contributions of Th2 cytokines to LN, which include the production of IL-6 and IL-4 by activated basophils that leads to autoantibody deposition in the kidney via enhanced Th2 response and B cell activation [9]. Another study reported that serums IL-18, IL-4 and IL-17 were identified as reliable biomarkers in type-IV lupus nephritis patients [4]. Hitherto, the treatment of SLE relies largely on the usage of broad-based steroids with adverse side effects. Thus, organ-specific tissue damage-inducing cytokines may be an ideal, targeted therapy for better outcome in SLE patients.

Interleukin-25 (IL-25), which is also known as IL-17E, is a cytokine belonging to the IL-17 family. IL-17 family consists of five other members, including IL-17A, IL-17B, IL-17C, IL-17D, and IL-17F [10]. Nevertheless, IL-25 appears to be different from others in terms of its structure and function. It typically asserts its function in allergic and humoral responses and is elevated in diseases, such as asthma, multiple sclerosis, rheumatoid arthritis (RA), and systemic lupus erythematosus (SLE) [11]. Meanwhile, IL-25 is considered as a Th2 cytokine that is capable of enhancing T-helper cell type-2 inflammatory responses. Furthermore, IL-25 induces Th2 inflammatory reactions and modulates expression of Th2-associated cytokines, such as IL-4, IL-5 and Il-13 [12]. Moreover, the expression of IL-17BR, a receptor for IL-25, is also potentiated by Th2 cells *in vivo* [13]. On the other hand, the serum level of IL-25 is shown to be elevated in SLE patients compared to healthy controls, and its level is highly associated with disease activity [14]. In addition, IL-25 was shown to generate IL-8 cytokine in kidney cells by stimulating the transcription factor, NF-κB [15]. Involvement of IL-25 and its role in stimulating Th2 cytokines in SLE and SLE-associated lupus nephritis remains obscure. Hence, the present study was designed to examine several novel cytokines from Th2 subsets, which may be involved in the pathogenesis of SLE and SLE-LN among Malaysian female patients. Evidence has documented that IL-25 injection in mice induces the expression of IL-5, which is a Th2-associated cytokine [16]. Thus, a correlation analysis was also conducted to elucidate the involvement of IL-25 in inducing the Th2 cytokines in all SLE patients.

## Methodology

### Subjects

In this study, sixty-four (64) Malaysian adult patients diagnosed with SLE and aged between 16–45 years old were recruited from Hospital Serdang, Selangor, Malaysia. The inclusion criteria for patient’s recruitment were: patients of 16 years old and/or above, Malay, females diagnosed with SLE according to the established SLE criteria by the American College of Rheumatological Association [17], and those who have consented their participation in this study. The exclusion criterion was patients who are pregnant.

In this study, fifteen (15) patients with their gender and age that matched to a healthy subject (population-based control) were free from clinical evidence or family history of any autoimmune disease based on their clinical history and physical examinations. An informed consent was obtained from each participant prior to their blood withdrawal. The recruitment period for the patients and healthy subjects was from January 2016 to October 2016. All patients and healthy controls recruited in the study were not related to each other and their ethnic background was assessed by self-description based on questions on ancestry. The patients were defined as Malay ethnicity only if both his or her parents are Malays. This study was approved by the institution’s Ethics Committee for Research Involving Human Subjects, Universiti Putra Malaysia; and the Medical Research and Ethics Committee, Ministry of Health Malaysia (NMRR-14-1756-23234).

### Disease categories

SLE patients were categorised as having lupus nephritis (SLE-LN), i.e., with renal involvement (N = 17) and/or without renal involvement (N = 47). Patient classification was based on the physical examination and clinical data available for a minimum of 5-year follow-up period. Active nephritis was diagnosed according to the clinical features of renal disease/flare and the International Society of Nephrology/Renal Pathology Society (ISN/RPS) 2003 Classification of Lupus Nephritis. The clinical features include increased proteinuria (> 2 gm), presence of red cells, casts in urine, increased creatinine, low C3, and rising anti-dsDNA antibody and ESR [18].

### Disease activity and damage scoring

The SLE disease activity was scored using Systemic Lupus Erythematosus (SLE) Disease Activity Index (SLEDAI) at the point of blood withdrawal [19]. Patients who had SLEDAI score of below 3 were considered as mild; while those with scores between 3–12 and above 12 were categorized as moderate flare and severe flare, respectively [20]. Active SLE was defined as SLEDAI score > = 10, while patients with SLEDAI < 10 were categorised as inactive [21]. SLE patients with lupus nephritis were determined based on confirmed renal histopathology by nephrologist based on the International Society of Nephrology/Renal Pathology Society (ISN/RPS) 2003 Classification Criteria of Lupus Nephritis.

### Blood sample collection

The blood samples from each fasting patient that engaged in this study were put into EDTA tubes (BD Lifesciences, UK). Within an hour of their collection, the blood samples were centrifuged at 2,000 g to isolate the plasma. Then, the plasma was immediately stored at -80°C until it is needed for cytokine analysis. The samples were collected before any additional dosage of corticosteroids, immunosuppressive drugs or introduction to antimalarial drugs for the recruited patients in both categories.

Laboratory investigations, including routine blood testing, full blood count, ANA, C-Reactive Protein (CRP), complement C3, C4, anti-dsDNA, immunoglobulin E (IgE), and urinalysis on the patients’ urine, were extracted from the Hospital Serdang Information System.

### Cytokine analysis

The cytokine levels of IL-4, IL-5, IL-6, IL-10, and IL-13 were measured using multiplex (R&D Systems, USA), which is a bead-based multianalyte profiling kit for detecting cytokine in plasma. Levels of cytokine IL-9 and IL-25 expression were detected using the enzyme-linked immunosorbent assay (ELISA) according to the manufacturer’s instructions (EIAab Science Co., Ltd., China). Data were expressed as mean ± standard error (mean ± SEM).

### Statistical analysis

Statistical analysis of numerical data was carried out by using GraphPad Prism statistical software for Windows, version 5.0 (GraphPad Prism Inc., USA). Kruskal-Wallis test, followed by post-hoc Duncan analysis, was used to compare the differential expression of cytokine between SLE with LN, SLE without LN, and control groups. Spearman’s rank correlation analysis was conducted to determine the associations between IL-25 and Th2-associated cytokine concentrations, as well as with the SLEDAI score, CRP, C3, C4, IgE levels, RBC, hematocrit, platelet, protein, creatinine, ESR, basophil, and urinalysis. A p-value of less than 0.05 was considered statistically significant.

## Results

### 1. Characteristics and disease activity of SLE patients

Forty-seven (47) SLE patients without lupus nephritis (LN), seventeen (17) SLE patients with lupus nephritis (LN) and fifteen (15) age- and sex-matched and control participants were studied. All subjects were Malaysian Malay females. The age, duration of diagnosis, duration of the disease, time since diagnosis with SLE-LN, presence of ANA and anti-dsDNA, and SLEDAI scores for SLE patients with and without lupus nephritis (LN) are summarized in Table 1.

**Table 1:** Characteristics of SLE patients and control groups.

Category	SLE (SLE without LN)	SLE-LN (SLE with LN)	Control
Number of subjects (N)	47	17	15
Age (Median ± SEM)	29.00 ± 0.799*	27.00 ± 1.404	27.00 ± 0.949
Antinuclear Antibodies (ANA)	Active (n = 40)	All active	n/a
	Non-active (n = 7)	n/a	
Anti-dsDNA	Active (n = 30)	All active	n/a
	Non-active (n = 17)		
**SLEDAI Activity Score**			
Mild (0–3)	-	-	n/a
Moderate (3–12)	9.200 ± 0.3916 (n = 25)		n/a
Severe (>12)	21.23 ± 2.044 (n = 22)	29.47 ± 2.011(n = 17)	n/a

*Results expressed as significantly different p < 0.05 from control group

n/a: not-applicable

SEM: Standard Error Mean

All SLE patients with LN presented severe activity, whereas for SLE patients without LN, 25 out of 47 had moderate activity and the remaining 22 of them had severe activity. All SLE patients with LN were presented with positive anti-nuclear antibodies (ANA) and positive anti-double stranded deoxyribonucleic acid. Moreover, patients with LN were presented with urinary casts, hematuria and proteinuria. LN was confirmed only by nephrologist based on the patient’s renal biopsy

### 2. Clinical presentations of SLE patients with and without lupus nephritis (LN)

**Fig 1:**
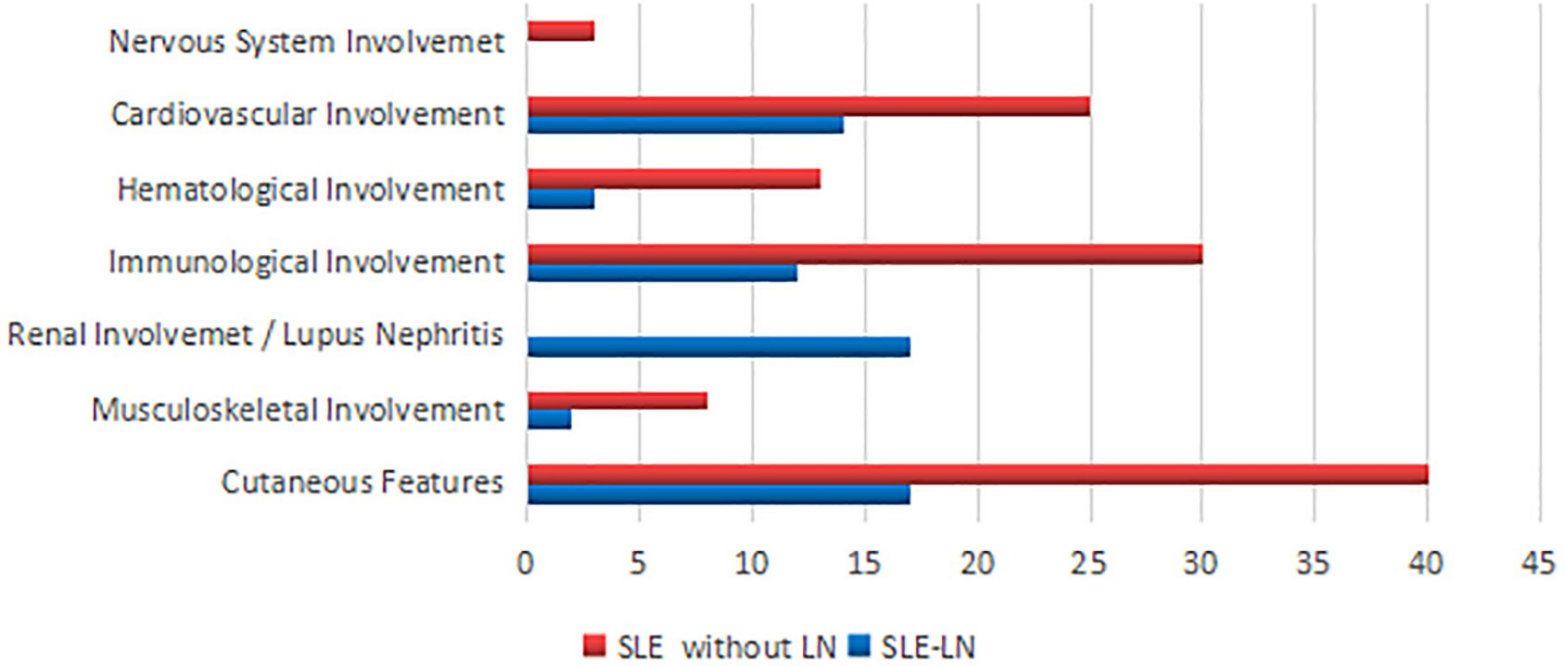
Percentage of SLE patients with LN (SLE-LN) and SLE patients without LN (SLE) that presented various clinical manifestations and/or other complications.

The clinical presentations of SLE patients with and without lupus nephritis (LN) are shown in Fig 1. The majority of SLE patients with and without lupus nephritis (LN) were reported to have malar rash, photosensitivity, as well as cardiovascular, immunological and hematological disorders. Only SLE patients with lupus nephritis (LN) showed renal involvement.

### 3. Assessment of severity markers in the blood of SLE-LN patients versus SLE patients

Levels of C3, C4, CRP, and IgE are presented in Table 2 below. The level of C3 in SLE-LN was the lowest, with 0.68 g/L, and that of the SLE group without LN was of 0.89 g/L. Similarly, the level of C4 was found to be the lowest in SLE-LN, followed by SLE without LN. On the other hand, the level of CRP was noted to be significantly higher (p < 0.0001) in SLE-LN than in SLE without LN. IgE also showed a significantly increased level (p < 0.0001) in the SLE-LN group, with 348.4 IU/mL; whereas that of the SLE without LN was of 158 IU/mL.

**Table 2:** Levels of C3, C4, CRP, and IgE in SLE without LN and SLE with LN.

Category	SLE (SLE without LN)	SLE-LN (SLE with LN)	Control	P-value
Complement (C3) (g/L) (Mean ± SD)	0.89 ± 0.07	0.68 ± 0.10	n/a	NS
Complement C4 (g/L) (Mean ± SD)	0.39 ± 0.029	0.30 ± 0.04	n/a	NS
C-Reactive Protein (CRP) (mg/L) (Mean ± SD)	1.91 ± 0.12	3.36 + 0.39	n/a	0.0003***
IgE (IU/mL) (Mean ± SD)	158 + 16.86	348.40 + 38.82	n/a	< 0.0001***

Mann-Whitney U test was used for comparison between SLE with and without LN.

***The results expressed were significantly different p < 0.001 between SLE with and without LN.

n/a: Not applicable

NS: Not significant

SD: Standard Deviation

**Fig 2:**
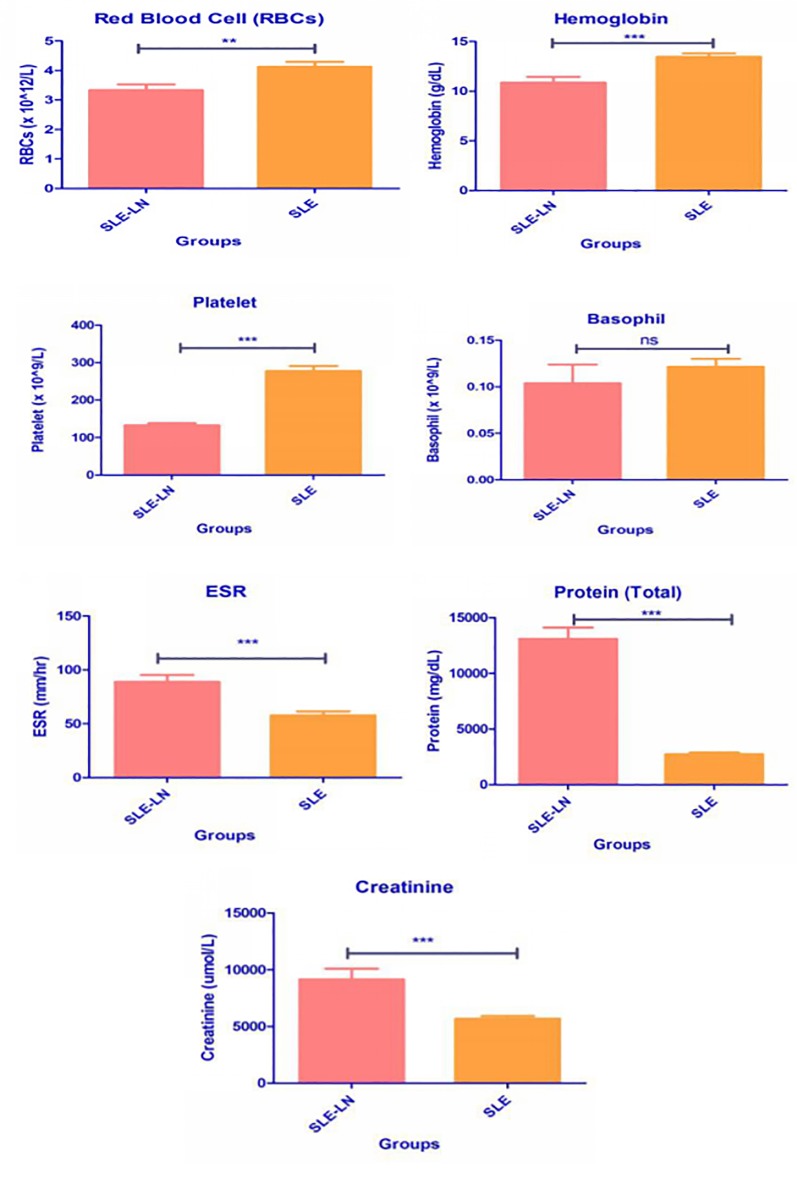
Levels of red blood cells (*×* 10^12^/L), hemoglobin (g/dL), platelet ((*×* 10^9^/L), Basophil ((*×* 10^9^/L), ESR (mm/hour), protein (mg/dL), and creatinine (umol/L).

Levels of red blood cells (×10^12^/L), hemoglobin (g/dL), platelet ((×10^9^/L), Basophil ((×10^9^/L), ESR (mm/hour), protein (mg/dL), and creatinine (umol/L) were also included in Fig 2.

Level of RBCs, hemoglobin and platelet were significantly lower (p < 0.0001) in the SLE-LN group, whereas ESR was significantly higher (p < 0.0001) in the SLE-LN group. Basophil expression was lower in the SLE-LN group, while protein and creatinine levels were significantly higher (p < 0.0001) in the SLE-LN group, as compared to the SLE without LN group.

### 4. Urinalysis among SLE-LN patients only

Additional measurement conducted on SLE-LN patients was urine analysis to detect the presence of blood, proteinuria and leucocytes in urine. The results are shown in Table 3.

**Table 3:** Urinalysis in SLE-LN patients.

Description	Blood	Proteinuria	Leucocytes
**Median** ± **SEM**	1.706 ± 0.2230	2.529 ± 0.2728	1.765 ± 0.2353

SEM: Standard error of the mean

### 5. Serum cytokine levels in SLE patients and control group

**Fig 3:**
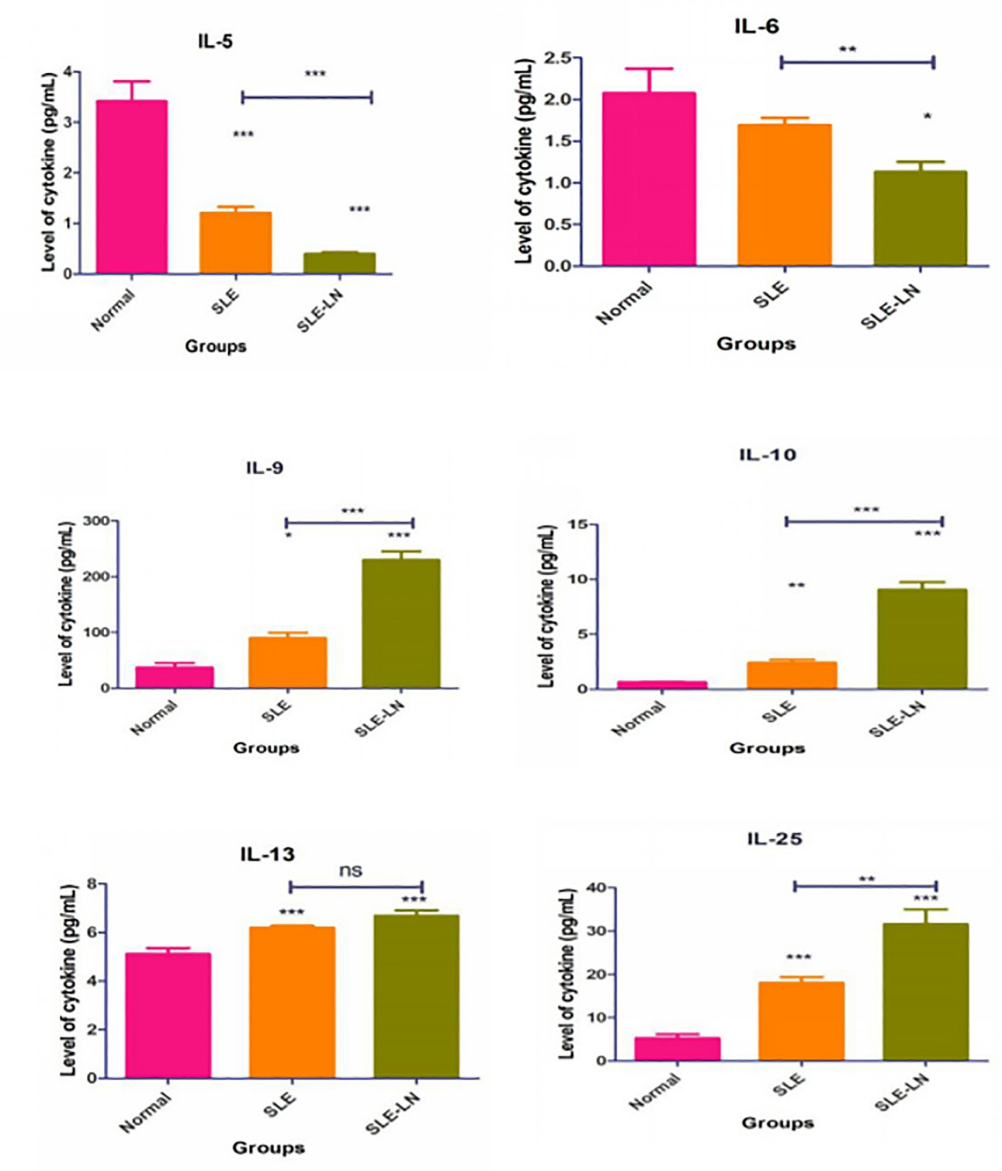
Mean expression levels of IL5, IL6, IL-9, IL-10, IL-13, and IL-25 in SLE and SLE-LN groups, as compared to the control group. Error bar indicates the comparison of the cytokine expression between the SLE without LN and SLE with LN groups. Kruskal-Wallis test, followed by Duncan analysis, were used to compare the differential expression of cytokines between the groups. Results were expressed as mean ± SEM. * indicates p < 0.05, **indicates p < 0.01, and ***indicates p < 0.001).

Both SLE-LN and SLE groups showed significantly higher levels of IL-9, IL-10, IL-13, and IL-25, but lower levels of IL-5 and IL-6 (p < 0.001) compared to normal control. Nevertheless, IL-4 was undetectable (result not shown). There was no significant difference of IL-6 in SLE without lupus nephritis, as compared to normal control. Only IL-9 and IL-25 were significantly increased in SLE-LN, compared to the SLE without LN and control groups (P < 0.001). The overall expression of these cytokines is depicted in Fig 3.

### 6. Correlation of serum cytokine levels with C3, C4, C-reactive protein (CRP), IgE, other renal markers, and SLEDAI in SLE-LN and SLE without LN groups

Correlation between serum cytokines IL-25, IL-5, IL-6, IL-9, IL-10, and IL-13 in SLE patients with and without LN with IgE, RBC, hemoglobin, platelet, protein, and creatinine is shown in Table 4. Correlation between cytokines to the renal flares parameter is shown in Table 5 and other correlations between cytokines, C3, C4, and CRP to SLEDAI and to cytokines are presented in Table 6.

**Table 4:** Correlation analysis between serum cytokines IL-25, IL-5, IL-6, IL-9, IL-10, and IL-13 in all SLE-LN patients (N = 17) with IgE, RBC, hemoglobin, platelet, basophil, ESR, protein, and creatinine.

	IgE	RBC	Hemoglobin	Platelet	Basophil	ESR	Protein	Creatinine
IL-25 Coefficient p-value	0.158 0.213	-0.999** 0.000	0.500* 0.041	-0.513* 0.035	-0.998** 0.000	0.996** 0.000	0.998** 0.000	0.437 0.079
IL-5 Coefficient p-value	-0.331** 0.007	0.980** 0.000	0.471 0.056	0.481 0.050	0.983** 0.000	-0.976** 0.000	-0.983** 0.000	-0.507* 0.038
IL-6 Coefficient p-value	-0.212 0.092	0.996** 0.000	0.450 0.070	0.473 0.055	0.996** 0.000	-0.995** 0.000	-0.996** 0.000	-0.405 0.107
IL-9 Coefficient p-value	0.465** 0.000	-0.997** 0.000	-0.529* 0.029	-0.549* 0.022	-0.996** 0.000	0.994** 0.000	0.996** 0.000	0.456 0.066
IL-10 Coefficient p-value	0.363** 0.003	-0.999** 0.000	-0.495* 0.043	-0.517* 0.033	-1.000**	0.998** 0.000	1.000**	0.431 0.084
IL-13 Coefficient P-value	-0.056 0.660	-0.288 0.262	-0.161 0.536	-0.183 0.482	-0.300 0.243	0.287 0.264	0.300 0.243	0.316 0.216

Spearman’s Correlation Test

**The results expressed were significantly different p < 0.01

*p < 0.05.

**Table 5:** Correlation analysis between serum cytokines IL-25, IL-5, IL-6, IL-9, IL-10, and IL-13 in all SLE-LN patients (N = 17) with the renal flares parameter.

		Blood	Proteinuria	Leucocytes
IL-25	Coefficient	-0.443	-O.589*	-0.441
	*p*-value	0.075	0.013	0.077
IL-5	Coefficient	0.439	0.559*	0.428
	*p-*valu*e*	0.078	0.020	0.087
IL-6	Coefficient	0.470	0.609**	0.478
	*p*-value	0.057	0.009	0.052
IL-9	Coefficient	-0.418	-0.561*	-0.417
	*p*-value	0.095	0.019	0.096
IL-10	Coefficient	0.456	-0.594*	-0.459
	*p*-value	0.066	0.012	0.064
IL-13	Coefficient	-0.346	-0.118	-0.237
	*p*-value	0.173	0.652	0.360

The presence of blood, proteinuria and leucocytes in the urinalysis is also correlated with serum cytokines. The correlation test was performed by using the Spearman’s rank correlation analysis.

* indicates P < 0.05; while

** indicates P < 0.01

**Table 6:** Correlation analysis between C3, C4, CRP, and serum cytokines IL-25, IL-5, IL-6, IL-9, IL-10, and IL-13 in all SLE patients with LN (N = 17) with SLEDAI score (SLEDAI > 3) and the correlation of C3, C4 and CRP with cytokines.

		SLEDAI	C3	C4	CRP
C3 (0.5–0.9 g/L)	Coefficient	-0.991**			
	p-value	0			
C4(0.1–0.4 g/L)	Coefficient	-0.831**			
	p-value	0			
CRP(0–5 mg/L)	Coefficient	-0.993**			
	p-value	0			
IL-25	Coefficient	0.994**	-0.997**	0.843**	-0.996**
	p-value	0	0	**0.426**	**0.108**
IL-5	Coefficient	0.973**	0.985**	0.798**	0.973**
	p-value	0	0	0	0
IL-6	Coefficient	-0.994**	0.991**	0.818**	0.991**
	p-value	0	0	0	0
IL-9	Coefficient	0.993**	-0.997**	-0.842**	-0.998**
	p-value	0	0	0	0
IL-10	Coefficient	0.995**	-0.998**	-0.830**	-0.997**
	p-value	0	0	0	0
IL-13	Coefficient	0.265	-0.312	-0.236	-0.301
	p-value	0.304	0.223	0.362	0.241

Spearman’s Correlations Rank Test

**The results expressed were significantly different p < 0.01

Significant positive correlations were detected between SLEDAI scores and serums IL-9, IL-10 and IL-25 in the SLE-LN group. The same cytokines showed significant negative correlations to C3, C4, CRP, RBCs, platelet, basophil, and proteinuria; but displayed positive correlations to IgE, ESR and protein. A significant negative correlation was observed for IL-5 and IL-6 to SLEDAI. IL-5 and IL-6 showed significant positive correlation with C3, C4, CRP, RBC, basophil, and proteinuria. IL-13 was not correlated with any of the parameters tested.

IL-25 and IL-10 were positively correlated to the SLEDAI score, whereas IL-5 was negatively correlated to the SLEDAI score. Among all other parameters tested, IL-25 was positively correlated to C3 only. IL-5 was positively correlated to protein and creatinine levels. A significant positive correlation was observed between IL-10 and basophil, and significant negative correlation was observed with creatinine and also IL-10. However, no significant associations were observed for IL-6, IL-9 and IL-13 in the SLE without LN group.

The Spearman’s correlation test showed a significant negative correlation between SLEDAI scores and C3 (P < 0.001), C4 (P < 0.001) and CRP (P < 0.001) for all SLE-LN patients. Whereas, no correlations were observed for SLE patients without LN. The details of Spearman’s correlation test are shown in Table 6.

### 7. Correlation of IL-25 with Th2 cytokines in all SLE patients

The relationship between IL-25 and T-helper 2 (Th2) cytokines was examined. As shown in Table 7, IL-9 and IL-10 showed a significant positive correlation with IL-25 in 64 patients of SLE with and without lupus nephritis.

**Table 7:** Correlation of IL-25 with Th2 cytokines in all SLE patients (N = 67).

	IL-5	IL-6	IL-9	IL-10	IL-13
IL25					
Coefficient	-0.050	-0.141	0.310*	0.498**	0.237
P-value	0.694	0.267	0.013	0.000	0.059

The correlation test was performed by using Spearman’s rank correlation analysis.

* indicates p < 0.05 while

** indicated p < 0.01

The relationship between IL-25 and T helper 2 (Th2) cytokines was examined on SLE-LN patients only, as shown in Table 8. IL-9 and IL-10 showed a significant positive correlation with IL-25 in all 17 SLE patients with lupus nephritis, whereas a significant negative correlation was observed with IL-5 and IL-6.

**Table 8:** Correlation of IL-25 with Th2 cytokines in all SLE-LN patients (N = 17).

	IL-5	IL-6	IL-9	IL-10	IL-13
IL-25					
Coeffcient	-0.981**	-0.994**	0.997**	0.998**	0.289
p-value	0.000	0.000	0.000	0.000	0.261

The correlation test was performed by using Spearman’s rank correlation analysis.

** indicates p < 0.01.

The relationship between IL-25 and T helper 2 (Th2) cytokines was examined on SLE patients without LN, as shown in Table 9. IL-10 showed a significant positive correlation with IL-25 in all 47 SLE patients without lupus nephritis, whereas a significant negative correlation was observed with IL-5.

**Table 9:** Correlation of IL-25 with Th2 cytokines in all SLE patients without LN (N = 47).

	IL-5	IL-6	IL-9	IL-10	IL-13
IL-25					
Coefficient	-0.387**	0.074	-0.011	0.340*	0.302
p-value	0.007	0.623	0.943	0.019	0.039

The correlation test was performed by using Spearman’s rank correlation analysis.

** indicates p < 0.01

## Discussion

An increasing number of evidences have shown that different cytokines may play different key roles in SLE patients [22]. However, the key role for each individual cytokines and their association to disease activity, disease severity and relationship with other cytokines in SLE remains elusive. Hence, this study was undertaken to examine the association of IL-25 and Th2 cytokines in SLE patients with and without lupus nephritis (LN) involvement among Malaysian Malay female patients. Previous studies have reported that females are more susceptible to SLE [23, 24, 25] than males due to variations in sex chromosomes and hormones. Besides that, the disease severity was generally higher among the Malays in Malaysia [25].

Complement activation and presence of autoantibodies are the indicators of SLE. Therefore, laboratory assessment for complement proteins, such as C3, C4, C3a, C5a, C3d, C4d, C-reactive protein, and autoantibodies, such as anti-nuclear antibody (ANA), anti-dsDNA, antinucleosome, and anti-C1q, were used extensively to measure the disease activity and severity in SLE. The findings of this research showed that all SLE patients with LN have positive ANA and anti-dsDNA. Meanwhile, for SLE patients without LN, 40 of them have active ANA and 30 patients have active anti-dsDNA. IgE, also known as immunoglobulin E, is an antibody associated with Th2 activities. Most studies have reported on its role that is mainly correlated with allergy reactions; however, several studies have shown that IgE plays a pathogenic role in autoimmune conditions, including SLE [26]. It has been reported that IgE autoantibodies to dsDNA have a putative role in the development of lupus nephritis [27]. The suggested mechanism is via basophils activation caused by the cross-linking of surface-bound IgE or IgE with immune complex in dendritic cells, which leads to the generation of Th2 cytokines [28].

A study by Narayanan et al. [29] have reported significantly high levels of anti-dsDNA, with lowest C3 and C4 levels in renal flare cases, as compared to non-renal flare cases. In addition, SLEDAI was positively correlated with anti-dsDNA and negatively correlated to C3 and C4 levels in renal flares, whereas no significant association was found among non-renal flare cases. This study also proved the same fact, whereby the Spearman’s rank correlation used in this work was stronger in the SLE-LN group.

Basophils are one of the unique immune cells having an impact on the adaptive immunity and potential to produce Th2 cytokines, besides mediating the development of lupus nephritis. A study on mice has reported that the deficiency of basophil had resulted to reduction in ANA, causing plasma cell depletion in the spleen and its involvement in increasing the autoantibody production, thus leading to renal damage [30]. Elevated total IgE in SLE patients without allergy and study on murine model suggest that play a crucial role in lupus nephritis. Thus, IgE can be considered as having an association with the disease severity in SLE [31]. In this study, the level of IgE was significantly high in the SLE-LN group, which suggests its involvement in renal damage, followed by a significant negative correlation to IL-5 and IL-6 and positive correlation to IL-25, IL-9 and IL-10.

IL-25, a Th2 cytokine, signals through its heterodimeric receptor (IL-25R), which consists of IL-17RA and IL-17RB. It is involved in promoting type 2 inflammation via the constituent of signaling cascade, the epithelium and activated T-cells [12]. Furthermore, various studies have proven its role in infectious and inflammatory diseases. Moreover, IL-25 has the capability to induce Th2-associated cytokine production, including that of IL-4, IL-5, IL-9, and IL-13 [10, 32, 33].

This study showed that the IL-25 was significantly increased in all SLE patients, as compared to control group, in which the latter has higher level of IL-25 recorded in SLE-LN patients. This implies the involvement of IL-25 in the immunopathogenesis of SLE that was further illustrated by its positive correlation with disease activity. Additionally, IL-25 showed a positive correlation with IL-9 and IL-10 in all SLE patients, indicating the interplay between these cytokines might have a positive immunopathological impact on SLE. A recent study by Dalia et al. (2018) [12] also showed a positive involvement of IL-25 in SLE patients, with high levels of IL-25 correlated to disease activity. Subsequently, this indicates the potential of IL-25 as a biomarker in SLE.

Besides IL-25, this study also showed significantly higher levels of IL-6, IL-9, IL-10, and IL-13; but lower levels of IL-5 in SLE patients. Meanwhile, the level of IL-4 remains undetectable. IL-5 has been well established for its critical role in orchestrating inflammatory responses in allergic diseases, specifically asthma. Gene expression study by Carneiro et al. (2011) [34] demonstrated that IL-5 was overexpressed in SLE patients with severe skin lesions, suggesting the involvement of IL-5 in the SLE pathogenesis. In contrast with the above findings, this study showed that IL-5 was significantly inhibited in SLE patients, especially on SLE-LN patients. Furthermore, a negative correlation with CRP and IgE was recorded. This could be due to the involvement of Th2 CD4^+^ cells in the pathophysiology of SLE with LN. It has been advocated that SLE is a Th2-mediated disease due to its specificity in producing autoantibodies specific for self-antigens [35].

IL-6 is a multifunctional cytokine, having pleiotropic action by inducing pro- or anti-inflammatory action. IL-6 is synthesised by monocytes and macrophages in response to other inflammatory cytokines. The biological role of IL-6 is to control the immune responses and hematopoiesis. IL-6 is one of the pioneer cytokines investigated in relation to lupus; this is mainly due to its close association to B lymphocytes. IL-6 cytokine expression is also significant in this study, with its expression lower in SLE-LN patients compared to SLE patients. However, there was no significant correlation of IL-6 with SLEDAI, C3, C4, CRP, and IgE. Other studies showed significant correlation between IL-6 and SLE, lupus nephritis, SLEDAI scoring, erythrocyte sedimentation rate (ESR), and C-reactive protein to IL-6 expression [36]. IL-6 has also been observed among patients with infection and serositis. Peterson et al. (1996) [37] reported that the serum IL-6 is not a predictor of SLE disease activity; however, urine IL-6 could be considered as a marker for active lupus nephritis.

In this work, IL-10 was significantly increased among SLE-LN and SLE patients. Apart from that, IL-10 also showed positive correlation to IL-25 in all SLE patients. Furthermore, IL-10 was significantly and positively correlated with the SLEDAI scoring and IgE, but was negatively correlated to C3, C4, CRP, RBC, hemoglobin, platelet, basophils, and proteinuria. IL-10 is one of the most potent cytokines, playing a vital role in SLE disease pathogenesis and in causing disease flare [38]. It is generated by activated monocytes and T-cells, which enhance the development of B-cell and immunoglobulin synthesis. Similarly, in another study, IL-10 is highly correlated with disease activity, SLEDAI, anti-dsDNA, C3, C4, and lymphopenia [39]. A recent study also supported a significant increase in serum IL-10 concentration that is associated with lupus activity among Han Chinese patients with SLE. Consequently, this led to the suggestion of using IL-10 as a serum marker to study the disease activity [40].

This study also demonstrated that the expression of IL-9 was significantly higher in SLE-LN group, followed by SLE group, when compared to controls. Previously, higher level of IL-9 was seen in rheumatoid arthritis (RA) and SLE patients than in the healthy group [41]. Additionally, in this study, IL-9 also showed a significant positive correlation with disease activity, protein, IgE, and ESR. IL-9 showed significant negative association to C3, C4, CRP, RBC, hemoglobin, platelet, proteinuria, basophil, and IL-25 levels in all SLE patients. Similarly, a study from Ouyang et al. (2012)[19] showed that the serum IL-9 level and CD4^+^IL-9^+^ T-cells percentage was positively correlated to the SLE disease activity index (SLEDAI) [19]. Moreover, the enhanced synthesis of IL-9 in RA patients was found to be positively correlated with the increased disease activity, ESR, number of tender joints, rheumatoid factor, C-reactive protein (CRP), and number of swollen joints [42]. Increased level of IL-9 production has led to the enhancement of immunoglobulin synthesis and other autoantibodies, including anti-dsDNA antibody deposition in kidneys and spleen, causing damages to these organs. IL-9 is likely to be involved in SLE pathogenesis and can be considered as a potential biomarker in monitoring this disease.

IL-13 is another immune-regulatory cytokine that is synthesised by activated Th2 cells, mast cells and NK cells. IL-13 exerts anti-inflammatory effects on monocytes and macrophages, besides blocking the synthesis of inflammatory cytokines, such as IL-1B, TNF-α, IL-6, and IL-8. In the present study, the IL-13 expression was significantly higher in the SLE-LN group as compared to the control group, but not significant when compared to the SLE without LN group. Brugos et al. (2012)[16] reported that the enhanced production of cytokine IL-13 level was significantly higher in lupus nephritis patients compared to patients with SLE without renal involvement and healthy group. Additionally, there was no significant association between IL-13 and disease activity and all other parameters tested for both groups of SLE with and without LN. Moreover, a comprehensive study on the role of IL-13 in SLE remains scarce. Hence, further research is warranted to elucidate the key role of IL-13 in SLE pathogenesis.

Generally, cytokines derived from Th2 cells must be increased in all SLE patients, as well as in SLE-LN patients. However, in the present investigation, it has been found that IL-5 was significantly lower in SLE-LN group, compared to the normal group. However, IL-4 was undetectable but had a high level of IL-25. There are various possibilities that could be associated to these variations. Cytokine IL-4 is primarily expressed by the mast cells, Th2 cells, eosinophils, and basophils [43]. Meanwhile, cytokine IL-5 is expressed by both hematopoietic and non-hematopoietic cells, including Th2 cells and granulocytes (basophils and eosinophils) [44]. One reason could be that the present investigation is only using blood plasma to quantify the particular cytokines; thus, it might not be a true reflection of the cytokine. Apart from that, SLE is a disease with various organ involvement, thus, the low expression of this cytokine could be associated to specific clinical manifestations. One study have reported that SLE patients with severe or extensive skin lesions showed an overexpression of IL-5 at keratinocytes, the component of epidermal cells [32]. The majority of patients in this research had malar rash and photosensitivity, which are not a severe form of skin problem. Finally, the possibility of prednisone drugs may have contributed to the decrease in cytokine gene expression in some subjects, in which this possibility cannot be ruled out [45].

One of the limitations in the present investigation is that this study only included Malay females with limited patient population. In Malaysia, the Malays are the dominant race, comprising 70% of the total population. Thus, based on the high epidemiology prevalence of SLE in females, the patient groups in this work only consist of Malay females.

## Conclusion

In conclusion, IL-25 is an emerging interleukin of interest since its clear association with the disease activity of SLE and SLE-LN. Its association with Th2 cytokine (IL-9 and IL-10) expressions would be a potential biomarker in monitoring and predicting disease severity, which could be used in the future for the development of diagnostic and therapeutic options for SLE patients.
